# In This Issue

**Published:** 1997

## CONCEPTS AND ISSUES IN COA RESEARCH

How many children of alcoholics (COA’s) are in the United States? Are COA’s at risk of becoming alcoholic themselves? Dr. Michael Windle explains how answers to these fundamental questions can vary widely, depending largely on how research studies are designed and interpreted. Although the number of COA’s and the prevalence of alcohol problems among them are not yet clearly established, COA status certainly does not guarantee the development of alcohol problems or other adverse effects. In fact, an expanding body of COA research has confirmed that numerous risk and protective factors exist, all of which influence whether COA’s will develop problems with alcohol at some point in their lives. Dr. Windle presents a conceptual model of the biological, psychological, and social risk factors that stem from a family history of alcoholism and shows how these factors may interact with stressful events to influence alcohol problems in COA’s. (pp. 185–191)

## THE EFFECTS OF PRENATAL ALCOHOL EXPOSURE

Children born to mothers who drank during pregnancy constitute a distinct subgroup among children of alcoholics because they were directly exposed to alcohol’s toxic effects while in utero. Prenatal alcohol exposure can result in long-term negative effects that range in severity, from full-blown, clinically defined fetal alcohol syndrome (FAS) to a milder constellation of alcohol-related birth defects, depending on the mother’s consumption level and drinking pattern during various stages of fetal development. Ms. Cynthia Larkby and Dr. Nancy Day cite epidemiological data on the prevalence of FAS and review research detailing the specific effects of prenatal alcohol exposure, including diminished physical growth, characteristic facial features, and central nervous system deficits. (pp. 192–198)

## PARENTING INFLUENCES ON THE DEVELOPMENT OF ALCOHOL ABUSE AND DEPENDENCE

Both alcoholic and nonalcoholic parents play pivotal roles in shaping their children’s drinking behavior. Parenting influences fall into two categories—alcohol-specific and non-alcohol-specific—write Drs. Theodore Jacob and Sheri Johnson. Alcohol-specific parenting influences include the modeling of parents’ drinking behavior, “thinking” positively about alcohol and the effect it will have (i.e., alcohol expectancies), the parent-child relationship, and the family environment. These influences likely affect children of alcoholics more strongly than children of nonalcoholics. In contrast, non-alcohol-specific influences, such as parent-child interactions that favor aggressive, antisocial behavior or parents with psychological disorders, similarly increase the risk for alcohol problems in children of both alcoholics and nonalcoholics. (pp. 204–209)

## A BEHAVIORAL-GENETIC PERSPECTIVE ON COA’S

Alcoholism in the parents is strongly correlated with alcoholism and other behavioral and psychiatric problems in their children. Behavioral genetic research, including adoption and twin studies, attempts to distinguish the genetic, the shared environmental, and the nonshared environmental influences that contribute to the behavioral similarities found between parents and their children. Dr. Matt McGue reviews the findings of several adoption and twin studies, which indicate that both genetic and environmental factors contribute to the parent and child’s potential for alcoholism. Dr. McGue also presents two models that help explain the joint effects of a genetic predisposition and environmental risk factors for alcoholism. (pp. 210–217)

## THE ROLE OF FAMILY INFLUENCES IN DEVELOPMENT AND RISK

Although children of alcoholics (COA’s) are at increased risk of developing alcoholism and other mental health problems, the majority of COA’s exhibit none of these problems. These diverse outcomes may be related to the variability that exists among alcoholic families with respect to inherited characteristics, such as severity of parental alcoholism and presence of parental psychological problems, as well as to factors associated with ethnicity and socioeconomic status. Drs. Deborah A. Ellis, Robert A. Zucker, and Hiram E. Fitzgerald explain how researchers and clinicians can differentiate between high- and low-risk alcoholic families based on the presence of accompanying parental psychological disorders, particularly antisocial personality disorder. High-risk families generally are characterized by the aggregation of several risk factors, and COA’s from these families are at highest risk for developing behavioral and emotional problems. (pp. 218–226)

## PHYSIOLOGICAL RESPONSES IN SONS OF ALCOHOLICS

Sons of alcoholics (SOA’s) differ from sons of nonalcoholics (non-SOA’s) in the way they respond to a range of stimuli. These contrasts may be correlated to an increased risk in SOA’s for developing alcohol problems, according to Dr. Peter R. Finn and Ms. Alicia Justus. For example, SOA’s tend to react to stressful stimuli more strongly than do non-SOA’s and to exhibit physiological responses (such as increased heart rates) that are associated with heightened tension and anxiety. Following alcohol consumption, however, SOAs’ reactions to both stressful and nonstressful stimuli are dramatically reduced, much more so than the responses of non-SOA’s. For a short period after drinking, SOA’s also may experience more of alcohol’s rewarding effects. In addition, SOA’s appear to be less impaired by alcohol—a predictor of future alcohol problems that could forewarn a significant vulnerability to alcoholism in SOA’s. (pp. 227–231)

## NEUROPSYCHOLOGICAL RESPONSES IN COA’S

Alcoholics have been shown to exhibit deficits in certain cognitive functions, such as information processing and problem-solving. Similar deficits also may occur in children of alcoholics (COA’s), even those who have never used alcohol. Based on this finding, researchers have hypothesized that specific cognitive deficiencies may be useful “markers” of a vulnerability to alcohol. Although existing evidence is inconclusive, Drs. Sara Jo Nixon and Laura J. Tivis discuss research investigating this hypothesis. The key question remains, however: If COA’s have preexisting cognitive problems that are similar to those of adult alcoholics, will they have a greater risk of developing alcoholism? (pp. 232–236)

## EVENT-RELATED POTENTIALS IN COA’S

Scientists are investigating whether people who are at risk for developing alcoholism, such as children of alcoholics, can be distinguished from those who are not at risk simply by observing differences in brain electrical activity. Much of the research in this area has centered on a specific brain wave called P3, which occurs in response to certain sensory (i.e., visual or auditory) stimuli. Alcoholics have been found to exhibit an abnormally low-energy P3; a similar response occurs in nonalcoholics from alcoholic families. Drs. Bernice Porjesz and Henri Begleiter discuss evidence that low-energy P3’s are determined genetically and predate the development of alcoholism. This deficiency may reflect an inefficiency in brain processing that may contribute to a predisposition to alcoholism. (pp. 236–240)

## BREAKING THE CYCLE OF ADDICTION

Children of alcoholics (COA’s) are at increased risk for developing problems with alcohol. A number of key prevention and intervention efforts are attempting to reduce this risk. Ms. Ann W. Price and Dr. James G. Emshoff explore several types of programs for COA’s, concentrating on short-term group programs that can be offered in schools. The authors conclude that intervention should include four basic components: alcoholism education, skill building in the areas of coping and social competence, social support, and healthy alternative activities. In addition, the authors explain how research findings could be better translated into everyday practice by adopting a model called “action research.” According to the authors, such a model could lead to improved prevention and intervention services for COA’s. (pp. 241–246)

## PSYCHOLOGICAL CHARACTERISTICS OF COA’S

More than 20 years ago, researchers first noted that children of alcoholics (COA’s) appeared to be affected by a variety of problems over the course of their life span. These problems included fetal alcohol syndrome, which is first manifested in infancy; emotional problems and hyperactivity, which occur during childhood; emotional problems and conduct problems, found during adolescence; and the development of alcoholism in adulthood. Although much has been learned over the ensuing two decades, a number of controversial research areas remain. As noted by Dr. Kenneth J. Sher, despite a common interest in COA’s, clinically focused literature and research focused literature have resulted in two distinct bodies of knowledge. Dr. Sher reviews important research results, with emphasis on findings generated by the alcohol-research community. Attention also is given to examining the empirical validity of concepts that have been advanced by several influential clinicians from the COA field. (pp. 247–254)

## A CRITICAL ANALYSIS OF COA RESEARCH

Amid the sea of literature published on children of alcoholics (COA’s) over the past few decades, how can research-supported evidence be distinguished from speculation and overgeneralization? To help gain perspective on the evolution of COA research, *Alcohol Health & Research World* invited a select panel of preeminent researchers to comment on the “state of the field.” In a roundtable discussion format, Drs. Laurie Chassin, Theodore Jacob, Jeannette L. Johnson, Marc A. Schuckit, and Kenneth J. Sher offer their unique perspectives on COA research—past, present, and future. From the vantage points of their different research backgrounds and interests, the panelists discuss the driving forces that have made COA research a “hot” field and give a glimpse into what led them personally to this area and the status of their current activity. (pp. 258–264)

